# Evaluation of Antioxidant Properties and Phenolic Composition of Fruit Tea Infusions

**DOI:** 10.3390/antiox2040206

**Published:** 2013-09-30

**Authors:** Saliha Şahin

**Affiliations:** Deparment of Chemistry, Faculty of Science and Arts, University of Uludag, Bursa 16059, Turkey; E-Mail: salihabilgi@uludag.edu.tr; Tel.: +90-224-294-1747; Fax: +90-224-294-1899

**Keywords:** antioxidant capacity, fruit tea, infusion, HPLC, phenolic compounds

## Abstract

The popularity of fruit tea is increasing in the world because of its antioxidant properties and attractive taste. The aim of this study was to determine and compare the antioxidant property and phenolic composition of 16 different fruit teas. The antioxidant property and total phenol content of fruit teas depending on the extraction condition (water temperature) were examined using the ABTS (2,2-azinobis[3-ethylbenzothiazoline-6-sulphonic acid]) method and the Folin-Ciocalteu method, respectively. The contents of total flavonoid and total anthocyanin of fruit teas was determined by using the UV/Vis spectrophotometric method. The phenolic composition was determined and quantified by using high performance liquid chromatography and photodiode array detection (HPLC-PDA). The highest total phenol content and antioxidant capacity were determined in pomegranate (I). The highest contents of total flavonoid and total anthocyanin were determined in peach (III) and blackberry (I), respectively. Chlorogenic acid, quercetin, myricetin, rutin, rosmarinic acid and ferulic acid were determined in fruit teas. A water temperature of 100 °C was the most effective to extract the highest contents of total phenols, total flavonoids, total anthocyanins and the highest antioxidant capacity in 16 different fruit teas. The purpose of this study was to determine the effect of water temperature on the extraction and quantify the various phenolic compounds in fruit teas by HPLC method for industrial application in producing the extracts.

## 1. Introduction

Fruits, herbs and aromatic plants are important sources of phenolic compounds such as phenolic acids, flavonoids and anthocyanins [1,2,3,4], which have considerable antioxidant properties *in vivo* and *in vitro* [5,6]. Natural antioxidants are substances that may protect human cells against the effects of free radicals produced. Free radicals can damage cells, and may play a role in heart disease, cancer and other diseases [3]. Therefore, the antioxidant compounds may play an important role in the prevention of certain diseases [7].

Recently, the studies about biologically active ingredients such as alkaloids and phenolic compounds in food and beverages have been performed [8]. Tea is one of the most consumed beverages in the world because of attractive aroma and special taste. Fruit teas and traditional herbal infusions are preferred in many European countries [8]. Fruit tea consumption is depending on the type of fruit and preparation. People may infuse fruit tea several times and prepare with water at different temperatures (cold or hot). Fruits or plants that are used to make the teas would be impacted by temperature and would differ from the integrity of the leaves of fruits or plants. It is interesting to determine and compare the antioxidant properties and phenolic composition of fruit tea because of their potential important health effects. However, there is no literature in our knowledge that shows antioxidant properties and phenolic composition of fruit tea depending on temperature.

The objective of this study was to investigate the total phenol, total flavonoid, and total anthocyanin contents in various fruit teas as well as their antioxidant capacity. The second objective of this study was to determine the effect of water temperature on the extraction and quantify the various phenolic compounds in fruit teas by HPLC method.

## 2. Experimental Section

### 2.1. Materials

Folin–Ciocalteau phenol reagent, gallic acid, ABTS (2,2-azinobis[3-ethylbenzothiazoline-6-sulphonic acid] diammonium salt) and myricetin were purchased from Fluka (Buchs, Switzerland). Trolox ([±]-6-hydroxy-2,5,7,8-tetramethylchroman-2-carboxylic acid), rosmarinic acid, quercetin hydrate, rutin and chlorogenic acid were purchased from Sigma–Aldrich (St. Louis, MO, USA). Ferulic acid, HPLC grade of methanol and formic acid were purchased from Merck (Darmstadt, Germany). All standard solutions were prepared in methanol.

### 2.2. Sample Preparation

Fruit teas purchased in a local market were prepared using aqueous extraction procedure to stimulate household brewing conditions. Fruit tea samples (3 g) were poured with 30 mL of deionized water heated to 20, 40, 70 and 100 °C and stirred with a magnetic stirrer for 3 min. Fruit tea infusions were separated from the solid matrix by filtration through sheets of qualitative filter paper. The infusions were used for the determination of the content of total phenol, total flavonoid and total anthocyanin, antioxidant capacity and HPLC analysis. The fruit tea infusions were further passed through 0.45-µm membrane filters before analytical procedures.

### 2.3. UV/Vis Spectroscopy

Spectrophotometric measurements were performed on a UV/Vis spectrometer (Varian Cary 50) equipped with 10 mm quartz cuvettes.

### 2.4. Total Phenol Content

Total phenol content in fruit tea infusions was determined spectrophotometrically according to the Folin–Ciocalteu method described in the literature [4]. The total phenol content was expressed as gallic acid equivalent (GAE) in milligrams of gallic acid per gram of dried fruit tea.

### 2.5. Total Flavonoid Content

Total flavonoid content in fruit tea infusions was determined spectrophotometrically according to the colorimetric method described in the literature [4]. The total flavonoid content was expressed as catechin equivalent (CTE) in milligrams of catechin per gram of dried fruit tea.

### 2.6. Total Anthocyanin Content

Total anthocyanin content of fruit tea infusions was determined by means of the pH differential absorbance method as previously described in the literature [4]. Total anthocyanin content was expressed as milligrams of cyanidin-3-glucoside per gram of dried fruit tea.

### 2.7. Antioxidant Capacity

Determination of antioxidant capacity of fruit tea infusions was performed by ABTS method described in the literature [4]. The antioxidant capacity was expressed as Trolox equivalent (TE) in milligrams of Trolox per gram of dried fruit tea.

### 2.8. HPLC Analysis of Phenolic Compounds

An Agilent 1200 HPLC system (Waldbronn, Germany) was used for determination of phenolic compounds in fruit tea infusions. Chromatographic separation conditions were described in our previous study [3]. The mobile phase consisted of 1% formic acid in water (solvent A) and acetonitrile (solvent B). Gradient conditions were as follows; 0–10 min 13% B, 10–20 min 41.5% B, 20–25 min 70% B, 25–35 min 10% B, total run time is 35 min. Flow rate was 0.5 mL/min and injection volume was 10 µL. An XBridge C18 (4.6 × 250 mm, 3.5 µm) column from Waters (Ireland) was used for chromatographic separation. The monitoring wavelengths were 360 nm for chlorogenic acid, quercetin, myricetin, rutin, rosmarinic acid and ferulic acid. Peaks were identified on the basis of comparison of retention times and UV-Vis spectra with standards of phenolic compounds.

## 3. Results and Discussion

### 3.1. Total Phenol, Total Flavonoid and Total Anthocyanin Contents

Total phenol, total flavonoid, and total anthocyanin contents were determined in 16 different fruit teas produced in Turkey. Total phenol, total flavonoid, and total anthocyanin contents varied greatly among fruit tea infusions.

**Figure 1:**
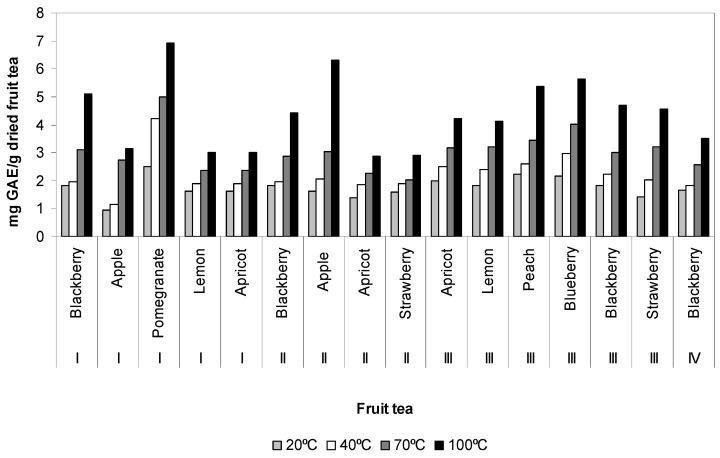
Total phenol content of fruit tea samples affected by temperatures.

Total phenol content of fruit teas ranged from 0.96 ± 0.01 to 6.91 ± 0.47 mg GAE/g dried fruit tea. Pomegranate (I) had considerably high total phenol content at water temperature of 100 °C and apple (I) had low total phenol content at water temperature of 20 °C. Total phenol content of fruit tea infusions affected by water temperatures (20, 40, 70 and 100 °C) are presented in Figure 1. Total phenol content of fruit tea infusions decreases at temperatures of 100 > 70 > 40 and 20 °C. As can be seen from Figure 1, total phenol content of fruit teas increases in the following order: pomegranate (I) > apple (II) > blueberry (III) > peach (III) > blackberry (I) > blackberry (III) > strawberry (III) > blackberry (II) > apricot (III) > lemon (III) > blackberry (IV) > apple (I) > lemon (I) > apricot (I) > strawberry (II) > apricot (II) at water temperature of 100 °C.

**Figure 2:**
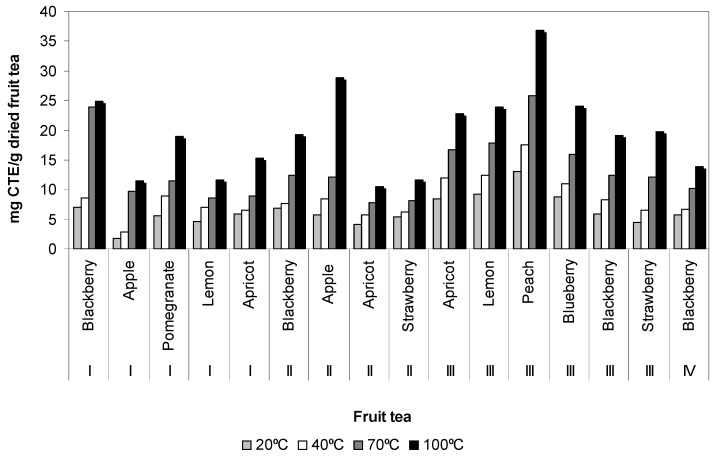
Total flavonoid content of fruit tea samples affected by temperatures.

Total flavonoid content varied greatly among the fruit tea infusions. The total flavonoid content varied from 1.70 ± 0.08 to 36.81 ± 0.10 mg CTE/g dried fruit tea. Peach (III) had the highest total flavonoid content among other fruit tea at water temperature of 100 °C (Figure 2). The total flavonoid content in apple (I) infusion at a water temperature of 20 °C was found to be lowest, similar to total phenol content. Total flavonoid content of fruit tea infusions decreases at temperatures of 100 > 70 > 40 and 20 °C. As can be seen from Figure 2, total flavonoid content of fruit teas increases in the following order: peach (III) > apple (II) > blackberry (I) > blueberry (III) > lemon (III) > apricot (III) > strawberry (III) > blackberry (II) > blackberry (III) > pomegranate (I) > apricot (I) > blackberry (IV) > strawberry (IV) > lemon (I) > apple (I) > apricot (II) at water temperature of 100 °C. The obtained total phenol content and total flavonoid content results confirmed the data in the literature [9] that at higher water temperature, total phenol content and total flavonoid content were higher and they reached to maximum values at 100 °C. Pomegranate (I) was recognized as the richest source of total phenol content, but peach (III) was recognized as the richest source of total flavonoid content. This indicates flavonoids, which are subgroups of phenolic compounds, had lower contribution to the total phenol content than the other phenolic compounds such as hydroxycinnamic acid, hydroxybenzoic acid, *etc*. The differences between total phenol content and total flavonoid content in blackberry (I, II, III, IV), apple (I, II), lemon (I, III), apricot (I, II, III), strawberry (II, III) could be due to environmental characteristics (for example; light, *etc*.).

**Figure 3:**
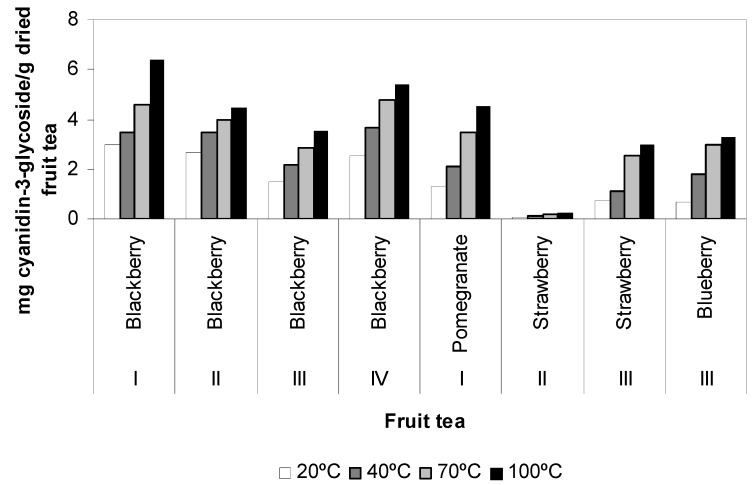
Total anthocyanin content of fruit tea samples affected by temperatures.

Total anthocyanin content was determined in eight different fruit teas that could be a richest source of anthocyanin: blackberry (I, II, III, IV), pomegranate (I), strawberry (II, III) and blueberry (III). Significant differences in total anthocyanin content were observed in fruit tea infusions (Figure 3). Blackberry (I) contained the highest anthocyanin, followed by blackberry (IV), pomegranate (I), blackberry (II), blackberry (III), blueberry (III), strawberry (III) and strawberry (II) at water temperature of 100 °C. Anthocyanins are water-soluble pigments. Water extraction resulted in significantly higher values for total anthocyanin content. The water temperature affected for total anthocyanin content. The results show that at higher water temperature, total anthocyanin content were higher and they reached to maximum values at 100 °C. Total anthocyanin content in fruit tea infusions was found to be higher at a water temperature of 100 °C than the berry extracts that were previously reported by literature [4].

### 3.2. Antioxidant Capacity

**Figure 4:**
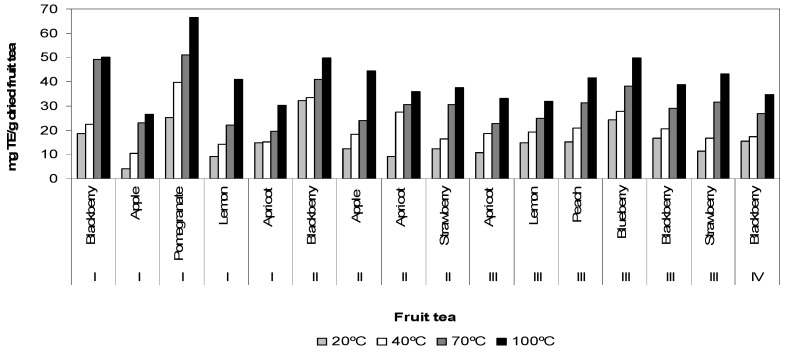
Antioxidant capacity of fruit tea samples affected by temperatures.

The antioxidant capacity of fruit teas was determined as milligrams TE/g dried fruit tea. The antioxidant capacity measured by ABTS assay ranged from 66.39 ± 0.99 to 4.08 ± 0.10 mg TE/g dried fruit tea (Figure 4). Pomegranate (I) exhibited the highest antioxidant capacity, followed by blackberry (I), blueberry (III), blackberry (II), apple (II), strawberry (III), peach (III), lemon (I), blackberry (III), strawberry (II), apricot (II), blackberry (IV), apricot (III), lemon (III), apricot (I) and apple (I). High level of antioxidant capacity in pomegranate (I) as compared to the other fruit tea could be due to its high level of total phenol content. Good correlation was found between total phenol content and antioxidant capacity (*r*^2^ = 0.78 at water temperature of 100 °C). The antioxidant capacities of fruit teas were found to be higher than that previously reported in the literature [10]. The results suggest that 78% of fruit tea antioxidant capacity results from the antioxidant capacity of phenolic compounds. However, the phenolic compounds are not only chemical substances that contribute the antioxidant capacity of fruit tea. Antioxidant capacity may come from the presence of other antioxidant secondary metabolites such as carotenoids, vitamins and volatile oils, which contribute to 22%. The results indicated that the antioxidant capacity of fruit tea infusions can be exerted mostly by the phenolic compounds. The phenolic compounds are important sources of natural antioxidants, which are known to reduce lipid peroxidation mediated deterioration of foods during processing, storage and play an important role in direct antioxidant capacity because of their scavenging ability due to their hydroxyl groups [11]. Antioxidants are substances that may protect human cells against the effects of free radicals produced when the body breaks down food, or by environmental exposures like tobacco smoke and radiation. The antioxidant compounds, such as phenolics, may play an important role in the prevention of certain diseases that are caused by free radicals.

There is no correlation (*r*^2^) found between total flavonoid content and antioxidant capacity of fruit tea infusions. This indicates that flavonoids had a lesser antioxidant capacity than phenolic compounds which played an important role in the antioxidant capacity of fruit tea infusions.

### 3.3. Determination of Phenolic Compounds

Phenolic compounds were determined in 16 fruit tea infusions prepared at 100 °C by HPLC-DAD. Chlorogenic acid, quercetin, myricetin, rutin, rosmarinic acid and ferulic acid were determined in fruit tea infusions (Table 1). Chlorogenic acid was detected in all fruit tea except lemon (I) with amounts ranged from 0.051 ± 0.003 to 8.265 ± 0.519 mg/g dried fruit tea. The highest amount of chlorogenic acid was established in strawberry (III). As can be seen from these results, the amounts of flavonoids such as quercetin and myricetin were detected in fruit tea with amounts ranging from 0.026 ± 0.001 to 0.057 ± 0.001 mg/g dried fruit tea and 0.082 ± 0.007 to 0.404 ± 0.012 mg/g dried fruit tea, respectively.

Rutin was quantitatively the major constituent of fruit tea infusion, except blackberry, apple and lemon (I), blackberry (II), apricot, peach, blueberry and blackberry (III), blackberry (IV) where it was not detected. The highest rutin content were found in fruit tea extracted at 100 °C, and decreased in following order: strawberry (III) > lemon (III) > strawberry (II) > pomegranate (I) > apricot (I) > apricot (II) > apple (II). Rosmarinic acid and ferulic acid were determined in fruit tea infusions with amounts ranging from 0.868 ± 0.001 to 1.347 ± 0.031 mg/g dried fruit tea and 3.619 ± 0.001 to 4.877 ± 0.017 mg/g dried fruit tea, respectively. Strawberry (II) contained the highest amounts of rosmarinic acid and ferulic acid. As rosmarinic acid is a known antiviral, antibacterial, antioxidant and anti-inflammatory [12], caffeic acid was found to have high activity comparable to that of quercetin [13] and ferulic acid was shown to inhibit the photo-peroxidation of linoleic acid at high concentrations [14]. Myricetin is a naturally occurring flavonoid, found in many grapes, berries, and other fruits. The researchers suggest that myricetin in high concentrations can modify LDL cholesterol and also it has antioxidant properties. The fruit tea may have antioxidant and anti-inflammatory properties due to quercetin, myricetin, rutin, chlorogenic, ferulic and rosmarinic acids contents [15,16]. The water temperature in household conditions usually ranges from 80 to 100 °C; it is clear now to mention that the highest total phenolic, flavonoid, anthocyanin contents, antioxidant capacities and phenolic compounds amounts were determined in fruit tea extracted at 100 °C.

**Table 1:** The amounts of phenolic compounds extracted from fruit tea (milligrams per gram dried fruit tea).

Sample	Fruit tea	CHA	QU	MR	RU	RA	FA
I	Blackberry	1.271 ± 0.010	0.035 ± 0.001	nd	nd	nd	nd
	Apple	1.025 ± 0.011	0.026 ± 0.001	nd	nd	nd	nd
	Pomegranate	0.051 ± 0.003	nd	nd	6.323 ± 0.006	nd	nd
	Lemon	nd	0.035 ± 0.005	nd	nd	nd	nd
	Apricot	1.976 ± 0.005	nd	nd	5.714 ± 0.012	1.195 ± 0.002	3.619 ± 0.001
II	Blackberry	1.485 ± 0.060	0.051 ± 0.001	nd	nd	nd	nd
	Apple	2.714 ± 0.020	nd	nd	5.614 ± 0.002	1.263 ± 0.005	4.321 ± 0.003
	Apricot	0.591 ± 0.031	nd	nd	5.632 ± 0.006	nd	nd
	Strawberry	8.265 ± 0.519	nd	nd	6.483 ± 0.029	1.347 ± 0.031	4.877 ± 0.017
III	Apricot	1.060 ± 0.001	nd	nd	nd	nd	nd
	Lemon	0.417 ± 0.001	nd	nd	6.746 ± 0.004	0.868 ± 0.001	nd
	Peach	1.028 ± 0.071	0.034 ± 0.001	nd	nd	nd	nd
	Blueberry	1.626 ± 0.021	0.036 ± 0.001	nd	nd	nd	nd
	Blackberry	0.897 ± 0.004	0.057 ± 0.001	0.082 ± 0.007	nd	nd	nd
	Strawberry	0.632 ± 0.027	nd	nd	8.566 ± 0.008	1.036 ± 0.007	nd
IV	Blackberry	0.860 ± 0.047	nd	0.404 ± 0.012	nd	nd	nd

Mean of two determinations ± SD. nd not detected, CHA chlorogenic acid, QU quercetin, MR myricetin, RU rutin, RA rosmarinic acid, FA ferulic acid, SD standard deviation.

## 4. Conclusions

Phenolic composition, total phenolic content, total flavonoid content, total anthocyanin and antioxidant capacity of 16 different fruit teas were determined in this study. This study provides information about phenolic composition and antioxidant capacity of fruit teas that may be consumed all over the world. Pomegranate (I) was recognized as the richest source of total phenolic content and antioxidant capacity, but peach (III) was recognized as the richest source of total flavonoid content. Total phenolic content, flavonoid content, anthocyanin and antioxidant capacity of fruit teas were higher by increasing the water temperature for the extraction and they reached maximum values at 100 °C. Phenolic compounds (chlorogenic acid, quercetin, myricetin, rutin, rosmarinic acid and ferulic acid) were determined in fruit tea infusion at water temperature of 100 °C by HPLC-DAD. Rutin was the most abundant phenolic compound determined in fruit teas.
